# Professor Frank Abbott

**Published:** 1897-11

**Authors:** 


### PROFESSOR FRANK ABBOTT.

WHEREAS, Death has removed from our ranks Professor Frank Abbott, of New York; and

WHEREAS, On account of his social qualities, his genial companionship, and his ability as a practitioner and teacher of dentistry, we realize the great loss to the profession in his death; therefore, be it

*Resolved*, That we tender to his family the sincere sympathy of this Association, and request that these resolutions be spread upon the minutes of the Association; and be it further

*Resolved*, That a copy of these resolutions be sent to the several dental journals of this country for publication.

